# A systematic review of the association between dietary patterns and health-related quality of life

**DOI:** 10.1186/s12955-020-01581-z

**Published:** 2020-10-12

**Authors:** Mahdi Vajdi, Mahdieh Abbasalizad Farhangi

**Affiliations:** 1 Research Center for Evidence Based Medicine, Health Management and Safety Promotion Research Institute, Tabriz University of Medical Sciences, Tabriz, Iran; 2 Drug Applied Research Center, Tabriz University of Medical Sciences, Attar Neyshabouri Street, Tabriz, Iran

**Keywords:** Health-related quality of life, Dietary patterns, Nutrition, Systematic review

## Abstract

**Background:**

Health related quality of life (HRQOL) is a potent indicator of individual’s happiness and life satisfaction. The way in which the HRQOL is affected by the diet is a topic of constant interest and debate among researchers. Evaluating the association between single nutrients or foods and HRQOL fails to take into consideration the complex interactions between nutrients. Also, the findings from previous investigations on the relationship between dietary patterns and HRQOL have been inconsistent. Therefore, our aim was to assess the existing evidence regarding the relationship between the dietary patterns and HRQOL by conducting a systematic review.

**Methods:**

A literature search was conducted in PubMed, Scopus, Web of Sciences and Google scholar databases from inception to March 2020, to identify studies that investigated associations between the dietary patterns (regardless of methods used to define dietary patterns) and HRQOL domains. Two researchers independently checked titles and abstracts, evaluated full-text studies, extracted data, and appraised their quality using the Newcastle–Ottawa Scale (NOS).

**Results:**

Thirteen studies (four longitudinal, and nine cross-sectional studies), with a total of 43,445 subjects, were included. Of the studies included in this review, eight studies evaluated the association between "Mediterranean" dietary patterns (MDP) and HRQOL, while five studies examined the association between different dietary patterns ("Healthy", "Unhealthy", "Western", "Fruit and vegetable", "Bread and butter" and etc.) and HRQOL. Excluding three studies which showed no significant association, healthy dietary patterns such as MDP, "Healthy" and "Fruit and vegetable" dietary patterns were associated with better HRQOL in physical and mental components scores. The quality assessment of included studies according to NOS criteria were ranged between medium to high quality.

**Conclusion:**

According to the current evidence, "Healthy" dietary patterns and “Mediterranean” dietary patterns are associated with better dimension scores of HRQOL in both physical and mental summaries. While, unhealthy dietary patterns and "Western" dietary patterns are associated with lower scores of HRQOL. Further longitudinal studies are required to clarify the association between dietary patterns and HRQOL

## Introduction

In recent years, life expectancy has increased in most countries, resulting in an increased prevalence of persons living with disabilities and chronic diseases [1, 2]. The quality of life is a very complex concept and contains different psychological, physical, social, and cultural aspects of well-being and health-related quality of life (HRQOL) improvement is one of the most important aims of healthcare systems. HRQOL is a multidimensional concept, which subjectively measures an individual’s social, emotional, functional and physical well‐being [3]. HRQOL represents an individual's perception of how health affects a person's life quality and overall well-being and is measured with either specific questionnaires (e.g., Hospital Anxiety and Depression scale (HADS)) or generic one’s (e.g., the 36-item Short Form (SF-36), the 12-item Short Form (SF-12)) [4].

Various factors, such as economic dependence [5], living situations [6], and lifestyle factors such as physical activity [7], and dietary habits [8, 9] can affect HRQOL. Among them, healthy dietary habits play an important role in our state of mental and physical health and prevention and treatment of non-communicable diseases [10, 11]. It is well established that an unhealthy diet can cause a reduction in physiological function and increasing the risk of disease development [12, 13], that there is a significant association between diet and alterations in immune and cognitive functions [14] and consequently that an improvement in diet is an important factor in the improvement of physiological function [15]. For instance, previous studies have shown that greater adherence to the "Mediterranean" diet (MED) is associated with a significant improvement in general psychological and physical health [4, 11]. In another study by Amarantos et al. [8], it has been highlighted that “Good nutrition improves HRQOL by promoting health, preventing dietary deficiency disease, and ameliorating or averting secondary malnutrition that is caused by or associated with other disease” (Amarantos, 2001, p.1).

Beyond single foods or nutrients, the assessment of whole dietary patterns is likely to provide a better explanation of diet-health relations. It is well established that people do not eat isolated nutrients and instead consume meals containing of a diversity of foods with complex combinations of nutrients that are likely to be interactive [16]. Whole-of-diet analysis represent a wider picture of a combination of foods and nutrients, such as the synergetic, additive, and antagonist effect of the foods [17] and provide researchers the opportunity to account for the interactions between different nutrients [18, 19]. Thus, dietary patterns may be more predictive of HRQOL and disease risk than foods or nutrients in isolation. Dietary patterns are derived based on empirical approach using statistical methods including principal component analysis (PCA) or cluster analysis [16]. PCA create groups by intercorrelated dietary variables, while cluster analysis groups individuals into categories according to their reported mean consumptions of foods [20].

Few studies have examined the relationship between dietary patterns and HRQOL and their results are inconsistent and the majority of the studies in literature have been limited by cross-sectional study design. For example, studies by Mozzillo et al. [21], Holmes et al. [22] and Perez-Tasigchana et al. (UAM-cohort) [23] have not found any significant relationship between dietary patterns and HRQOL. However, some studies have reported the association of namely "Western" or "Unhealthy" dietary patterns with physical and mental chronic disease [24–26] and poor HRQOL [17, 27, 28]. Also, several studies reported that "Western" dietary pattern (increased intake of saturated fat and refined foods along with low intake of vegetable and fruits) is inversely associated with healthy factors such as immunity [29] and chronic diseases [30, 31]. While dietary patterns recognized in each study may be different from each other, some important characteristics of the healthy dietary pattern such as high intake of fruits, vegetables, legumes, seafood, whole grains, and low intake of refined grains, processed meat and sweetened foods have been suggested to be related to positive health benefits [32, 33]. Moreover, "Mediterranean" style dietary pattern (MEDP) is also associated with decreased risk of chronic disease and improved HRQOL [34–37]. This pattern particularly consists of the intake of non-refined cereals and products, vegetables, fruits, olive oil and non-fat or low-fat dairy products is a known primary preventive tool against chronic cardiovascular events [38–40]. Numerous evidences have demonstrated that "Mediterranean" dietary pattern (MDP) reduces cardiovascular risk, improves survival from coronary heart disease (CHD), improved glycemic control and decreased risk of type 2 diabetes [41].Given the conflicting results and lack of systematically reviewed publication of earlier studies, the aim of this study was to systematically review published data to evaluate the relationship between dietary patterns and HRQOL among general population without age or disease restrictions.

## Methods

### Search strategy

A systematic search was conducted using Web of Sciences, Scopus, PubMed and Google scholar databases to the studies evaluated the relationship between dietary patterns and HRQOL from.

inception to March 2020. No language restriction was used. In search strategy, we used a combination of the MeSH (Medical Subject Headings) terms including the following: (Diet OR dietary OR patterns OR factor analysis OR cluster analysis OR principal component analysis OR diet patterns OR diet pattern OR dietary patterns OR dietary pattern OR eating pattern OR food patterns OR eating patterns OR food pattern OR patterns) AND (Life Quality OR Quality of Life OR Health-Related Quality of Life OR health status OR HRQOL OR QOL OR EQ-5D OR EuroQol 5 Dimensions OR SF-12 OR Short-form 12 OR SF-36 OR Short-form 36 OR life qualities OR questionnaire). Further explanations about the search strategy are provided in Table 1. Moreover, hand-searching from reference lists of potentially eligible studies, previous reviews was carried out to retrieve additional studies. The protocol of the present review has been registered in the International prospective register of systematic reviews (PROSPERO) and its registration number is CRD173914. Furthermore, the ethics committee of Tabriz University of Medical Sciences has approved the study’s protocol (Registration number: IR.TBZMED.REC.1398.672).

**Table 1:** Search strategy and number of publications in each electronic database

Data base	Search strategy	Number of publications
PubMed	(Diet[Title/Abstract]) OR "Diet"[Mesh]) OR dietary[Title/Abstract]) OR patterns[Title/Abstract]) OR factor analysis[Title/Abstract]) OR principal component analysis[Title/Abstract]) OR diet pattern[Title/Abstract]) OR diet patterns[Title/Abstract]) OR dietary patterns[Title/Abstract]) OR dietary pattern[Title/Abstract]) OR eating pattern[Title/Abstract]) OR eating patterns[Title/Abstract]) OR food pattern[Title/Abstract]) OR food patterns[Title/Abstract])) AND (Life Quality[Title/Abstract]) OR "Quality of Life"[Mesh]) OR Quality of Life[Title/Abstract]) OR Health-Related Quality of Life[Title/Abstract]) OR HRQOL[Title/Abstract]) OR QOL[Title/Abstract]) OR EQ-5D[Title/Abstract]) OR EuroQol 5 Dimensions[Title/Abstract]) OR SF 12[Title/Abstract]) OR SF-36[Title/Abstract]) OR life qualities[Title/Abstract]) OR questionnaire[Title/Abstract]) OR Short-form 36[Title/Abstract]) OR short form 12[Title/Abstract])	390
Scopus	( ( TITLE-ABS-KEY ( *"* Diet *"*) OR TITLE-ABS-KEY ( *"*dietary patterns*"*) OR TITLE-ABS-KEY ( *"* factor analysis *"*) OR TITLE-ABS-KEY (*"* principal component analysis*"*) OR TITLE-ABS-KEY ( *"* diet patterns *"*) OR TITLE-ABS-KEY ( *"* diet pattern *"*) OR TITLE-ABS-KEY ( *"*dietary pattern*"*) OR TITLE-ABS-KEY ( *"* eating patterns *"*) OR TITLE-ABS-KEY ( *"*eating pattern*"*) OR TITLE-ABS-KEY ( "food pattern") OR TITLE-ABS-KEY ( *"* food patterns *"*) OR TITLE-ABS-KEY ( *"*food pattern*"*) AND ( ( TITLE-ABS-KEY ( *"* Life Quality *"*) OR TITLE-ABS-KEY ( *"* Quality of Life *"*) OR TITLE-ABS-KEY ( *"* Health-Related Quality of Life *"*) OR TITLE-ABS-KEY ( *"* HRQOL *"*) OR TITLE-ABS-KEY ( *"* QOL *"*) OR TITLE-ABS-KEY ( *"* EQ-5D *"*) OR TITLE-ABS-KEY ( *"* EuroQol 5 Dimensions *"*) OR TITLE-ABS-KEY ( *"* SF 12*"*) OR TITLE-ABS-KEY ( *"* SF 36*"*) OR TITLE-ABS-KEY ( *"* life qualities *"*)	406
Web of science	(("dietary patterns" OR "dietary patterns" OR "Diet" OR "diet pattern" OR "diet patterns" OR "eating patterns" OR "eating patterns" *OR* "food pattern" OR "food pattern" OR "principal component analysis" *OR* " factor analysis ") AND ( "Life Quality" OR " life qualities " OR "Quality of Life" OR “health related quality of life” OR “HRQOL” *OR "* QOL *"* OR *"* EQ-5D *"* OR *"* EuroQol 5 Dimensions *"* OR *"* SF 12*"* OR *"* SF 36*"*))	478

### Inclusion criteria

Studies were evaluated for eligibility using the inclusion and exclusion criteria in Table 2. The search results were uploaded into EndNote software (version X8, for Windows, Thomson Reuters, Philadelphia, PA, USA) and duplicates were removed. Therefore retrieved articles were merged and the review process has been facilitated. Two reviewers (MAF and MV) independently assessed the titles and abstracts of all studies identified in the search. Studies not meeting the eligibility criteria were eliminated. Furthermore, the reference lists of relevant reviews and of included articles were also checked for further studies. Full-texts of relevant articles were retrieved if meeting the eligibility criteria and findings were re-screened. Any discrepancies were discussed between the two authors. The patient/Population; Intervention; Comparator; Outcome (PICO) question was as follows: in human models (P), does healthy dietary pattern (I) compared to unhealthy dietary pattern (C), affect HRQOL (O)?

**Table 2:** Inclusion and exclusion criteria for studies

Inclusion criteria	Exclusion criteria
Original human observational studies (cross-sectional, case control or cohort studies)	Interventional studies, case series, systematic review/ meta-analysis, case reports
Studies assessing the relationship between dietary patterns and HRQOL in all age groups and different disease	Studies that did not report HRQOL as an outcome
Studies were included if they evaluated the HRQOL with a valid questionnaire including but not limited to: SF-12, SF-36, WHOQOL, EORTC QLQ*-*C 30*,* PedsQL 3.0DM	–
Studies that evaluated the dietary intake by FFQ, 24-h recall methods, food records or similar instruments	–
Studies that examined whole diet (regardless of methods used to define dietary patterns)	Studies that examined single nutrients, single foods, or single food groups

### Data extraction

Data extraction was conducted by two independent reviewers (MAF and MV), and any disagreements were resolved by consensus. The following data were extracted using a standard form: first author's name, study location, publication year, study design, sample size, age and gender of subjects, type of study population, dietary pattern assessment method, total number of participants and the number of case and controls, the HRQOL assessment tool and information about adjustments for possible confounders the main results.

### Quality assessment

The methodological quality of included studies was evaluated using the Newcastle–Ottawa scale (NOS) adopted for cross-sectional and cohort studies. The 9-point NOS scale has scoring ranges from 0 to nine [42]. The tool assesses the studies based on three dimensions-selection, compatibility, exposure or outcome. Both authors rated the article independently and discussed the ratings.

## Results

**Fig. 1:**
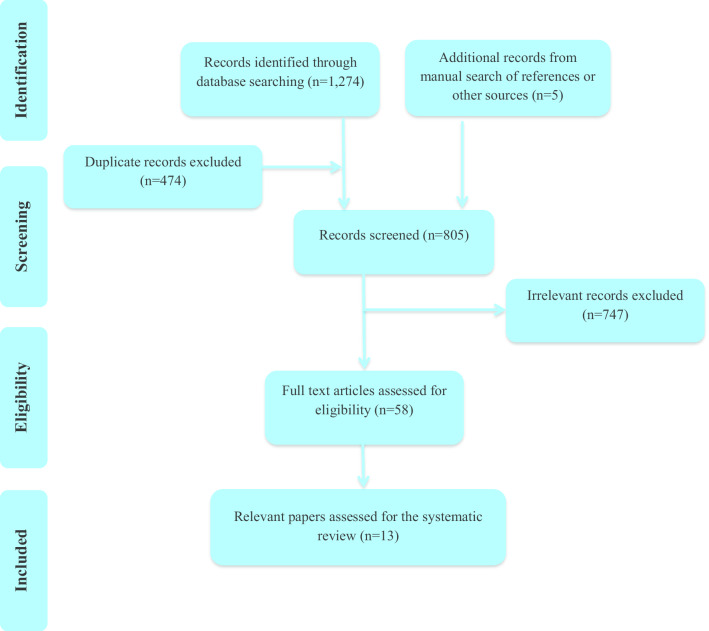
Flow diagram of search strategy and selection of studies

The current study follows the Preferred Reporting Items of Systematic Reviews and Meta-Analysis (PRISMA) guidelines for reporting the systematic reviews [43]. The flowchart of the study selection process is described in Fig. 1. A total of 1274 studies from four electronic databases and a further of five from hand searching were found. Removing 474 duplicates, 805 articles were screened for title and abstract review and 58 articles were eligible for full text review. Finally, excluding 45 papers because of not meeting the inclusion criteria –not assessing the dietary pattern with either factor analysis or MDP method, not assessing the statistical associations between dietary patterns and HRQOL, design of intervention- a total of thirteen articles were included in the current systematic review.

The characteristics of included papers are presented in Table 3. Two longitudinal study [27, 44], two cohort studies analyzing the baseline characteristics [23, 28] and nine cross-sectional studies [17, 21, 22, 34–36, 45–47] were included. Studies were published between 2016 and 2019 and seven of these studies were carried out in Spain [17, 23, 28, 34, 36, 44, 45], two in Italy [21, 35], one in France [22], one in USA [27], one in Australia [46] and one in Iran [47]. Studies where cross-sectional data were derived from longitudinal, cohort or even randomized trials were reported as cross-sectional. The largest sample size belonged to the study of Bonaccio et al. [35] considering 16,936 participants and the lowest samples size was belonged to the study of Gigic et al. [27] with 192 participants. The studies in the present review evaluated the relationship between dietary patterns evaluated with PCA or MDP score with HRQOL. We did not exclude disease status; accordingly five studies evaluated the dietary patterns with factor or cluster analysis [17, 22, 27, 28, 47], while eight studies evaluate the MDP [21, 23, 34–36, 44–46]. Moreover, among all of the studies, one study was carried out among breast cancer survivals [17], one among subjects with intermediate cardiovascular risk [44], one in colorectal cancer [27], one in multiple sclerosis [47], one among patients with type 1 diabetes [21], one among patients with type 2 diabetes [34], one in patients with minor digestive symptoms [22] and others among general apparently healthy individuals. From the perspective of age, two studies were among older individual [23, 45] while others were performed among general population [17, 21, 22, 27, 28, 34–36, 44, 46, 47].

**Table 3:** Characteristics of studies included in the systematic review owing to reporting the association between dietary patterns and health-related quality of life

First author	Year	Country	Design	Disease status	Sex	Age range	Total sample size	N. of cases / controls	Dietary pattern assessment method	HRQOL assessment method	Result	Adjusted variables
Mozzillo et al. [21]	2017	Italy	Cross-sectional	Participants with type 1 diabetes	Both	13 -19	242	110women/ 132 men	MED score by KIDMED (16 questions) score to assess adherence	Italian version of the PedsQL 3.0 DM	No significant associations between MED and quality of life were found	-
Moravejolahkami et al. [47]	2019	Iran	Cross-sectional	Multiple sclerosis patients	Both	20–60	261	210 women/ 51 men	Factor analysis with three dietary patterns ("Fruits, Vegetables, Low fat dairy-based dietary" pattern, "Mediterranean-Like" dietary pattern and "Western-Like" dietary pattern) by semi-quantitative FFQ	MSQOL-54	Fruits, Vegetables, Low fat dairy-based pattern and Mediterranean-Like pattern were associated with higher physical and mental health composite scores (P < 0.001)	Age, sex, type of multiple sclerosis and duration of the disease
Sanchez-Aguadero et al. [44]	2016	Spain	Longitudinal follow-up study	Subjects with intermediate cardiovascular risk	Both	35–74	314	159 women/ 155 men	MED score by FFQ	SF-12	Greater adherence to the MED was associated with higher scores on the SF-12 mental component, social functioning. 1.17 point increase in the mental component for each increase of 1 point in the MED adherence score (p < 0.01)	Age, sex, hypertension, dyslipidemia and Charlson Comorbidity Index
Zaragoza-Marti et al. [45]	2016	Spain	Cross-sectional	Elderly free-living	Both	More than 60	351	201 women/ 150 men	MDP by short FFQ (MEDIS-FFQ) validated for older adult	SF-12	Higher adherence to MED was associated with higher QOL. In adjusted model, MED was associated with PCS and MCS in men and with MCS in women	Age
Kim et al. [17]	2018	Spain	Cross-sectional	Breast cancer survivors	Women	12–79 years	232	58/58	Factor analysis with two major dietary pattern "Healthy" and "Western" assessed by non-consecutive 3-day dietary record on 2 weekdays and 1 weekend day	EORTC QLQ-C30) and (QLQ-BR23)	"Healthy" dietary patterns were associated with better scores for dyspnea but worse scores for insomnia among breast cancer survivors	Age, BMI, marital status, education level, cancer stage, physical activity, time since surgery and menopausal status
Gigic et al. [27]	2017	USA	Longitudinal follow-up study	Colorectal cancer patients	Both	≥ 18	192	58/58	Factor analysis with four major dietary pattern of "Western", "Fruit & vegetable", "Bread & butter" and "High- carbohydrate" by FFQ	EORTC QLQ-C30	Patients following a "Western" diet had lower chances to improve in physical functioning, constipation and diarrhea over 12 months post-surgery. Patients following a ''Fruit & vegetable" diet showed improving diarrhea scores	Sex, age, tumor stage, tumor site, and stoma
Holmes et al. [22]	2018	France	Cross-sectional	Subjects with minor digestive symptoms	Women	18–60 years	324	100/ 58	Factor analysis with four major dietary pattern of "Healthy", "Unhealthy", "Balance" and "Convenience" by non-consecutives 24 h recall method	Food Benefit Assessment questionnaire	No significant difference in none of dimensions of quality of life in different clusters was observed	Age
Perez-Tasigchana et al. [23]	2016	Spain	UAM-cohort (baseline data)	Community dwelling individuals	Both	≥ 60	2,376	594/594	MED was assessed using three approaches of MDP index, PREDIMED score and Trichopoulou’s MED score using FFQ	SF-36	No significant associations between the MDP and the PCS or the MCS were found	Sex, age, education, tobacco, BMI, abdominal obesity, hypertension, leisure-time physical activity, time spent watching TV, energy intake, diabetes, hypercholesterolemia, CHD, stroke, cancer, and depression
Perez-Tasigchana et al. [23]	2016	Spain	Seniors-ENRICA cohort (baseline data)	Community dwelling individuals	Both	≥ 60	1,911	478/477	MED was assessed using three approaches of MDP index, PREDIMED score and Trichopoulou’s MED score using FFQ	SF-12v.2	A higher PREDIMED score was associated with a slightly better PCS; when compared with the lowest tertile of PREDIMED score, the beta coefficient for PCS was 0.55 (−0.48, 1.59) in the second tertile, and 1.34 (0.21, 2.47) in the highest tertile. However, the PREDIMED score was non-significantly associated with a better MCS score. The MSD did not show an association with either the PCS or the MCS	Sex, age, education, tobacco, BMI, abdominal obesity, hypertension, leisure-time physical activity, and time spent watching TV, energy intake, diabetes, hypercholesterolemia, CHD, stroke, cancer, and depression
Alcubierre et al. [34]	2016	Spain	Cross-sectional	Patients with type 2 diabetes	Both	≥ 18	294	294	rMED by FFQ	ADDQoL-19	The adherence to the MED showed no significant association with the overall QOL score. However, rMED was associated with some HRQOL dimensions: travels, self-confidence and freedom to eat and drink	Adjusted for insulin treatment, retinopathy, diabetes duration, age (> 65 years), waist, ethnicity
Milte et al. [46]	2015	Australia	Cross-sectional	Old individuals	Both	55–65 years	2,457	516/887	MED diet score by FFQ	SF-36	MED score was positively associated with energy component of quality of life (OR = 1.53, CI = 1.11–2.10) only in women	Age, education, urban or rural location and menopausal status in women, smoking, physical activity and BMI
Ruano et al. [28]	2013	Spain	Cohort study (baseline data)	University graduates	Both	≥ 18	11,125	2,225/2,225	Factor analysis with four two dietary pattern of MDP score and "Western" dietary pattern by FFQ	Spanish version of the SF-36	"Western" dietary pattern was associated with lower HRQOL in all domains. The MDP was associated with better HRQOL domains	Age, sex, smoking, leisure time physical activity, total energy intake, baseline BMI and history of hypertension, diabetes, dyslipidemia, CVD
Bonaccio et al. [35]	2013	Italy	Cross-sectional	Community dwelling individuals	Both	≥ 35 years	16,936	4,234/4,234	Trichopoulou’s MED score by FFQ	SF-36	Mental health was associated positively with MED score, IMI and an "Olive oil and vegetable" pattern, but negatively with an "Eggs and sweets" pattern. Physical health was associated positively with MED score and "Olive oil and vegetable" pattern, but negatively with a "Meat and pasta" pattern	Age, sex, BMI, total energy intake, total physical activity, education, income, total socioeconomic status, smoking, diabetes, hypertension, hypercholesterolemia
Galilea-Zabalza et al. [36]	2018	Spain	Cross-sectional	Community dwelling individuals	Both	55–70 years	6,430	1,486/1,567	Traditional MDP score by 17-point questionnaire to assess adherence	Spanish version of HRQOL questionnaire	Higher adherence to the MED was independently associated with significantly better scores in the eight dimensions of HRQOL	Sex, age and recruitment center, BMI, physical activity, smoking status, marital status, highest level of education attained, high blood pressure, diagnosis of type-2 diabetes, history of depression, chronic lung disease, cancer

*FFQ* Food Frequency Questionnaire, *PedsQL 3.0 DM* Pediatric Quality of Life Inventory 3.0 Diabetes Module, *MSQOL-54* Multiple Sclerosis Quality Of Life-54 questionnaire, *EORTC QLQ*-*C30* European Organization for Research and Treatment of Cancer Quality-of-life Questionnaire Core 30, EORTC *QLQ*-*BR23* The EORTC Breast Cancer-Specific Quality of Life Questionnaire, *ADDQoL-19* Audit of Diabetes-Dependent Quality of Life, *IMI* Italian Mediterranean diet index, *CHD* coronary heart disease, *PREDIMED score* prevention with Mediterranean diet score *rMED* Relative Mediterranean diet score, *CVD* cardiovascular disease, PCS Physical component score, MCS Mental component score, *MEDP* Mediterranean style dietary pattern, *MDP* Mediterranean dietary pattern *MED* Mediterranean diet, HRQOL Health-related quality of life, SF-12, The 12-item Short Form, SF-36 The 36-item Short Form, EQ-5D The European Quality of Life-5 Dimensions, QOL Quality of Life, BMI Body Mass Index

Major variability was observed between the HRQOL assessment tools. Five studies [23, 28, 35, 44–46] measured HRQOL with standard questionnaires of SF-36, SF-12 reporting physical components scores (PCS) and mental components scores (MCS) along with scores obtained for eight domains. Studies which carried out among cancer patients used the European Organization for Research and Treatment of Cancer Quality-of-life Questionnaire (EORTC QLQ) for measuring HRQOL [17, 27], one study used Food and Benefit Assessment (FBA) questionnaire [22], One study carried out among multiple sclerosis patients used Multiple Sclerosis Quality Of Life-54 questionnaire (MSQOL-54) [47], one study used Pediatric Quality of Life Inventory 3.0 Diabetes Module (PedsQL 3.0 DM) (21), and one used Audit of Diabetes-Dependent Quality of Life (ADDQoL-19) [34]. Dietary assessment was also measured with variable tools. Most of the studies used food frequency questionnaires (FFQ) [23, 27, 28, 34, 35, 44–47], while one study evaluated dietary intake using three day non-consecutive food record [17], one study with non-consecutives 24 h recall method [22] and one with Traditional MDP score by 17-point questionnaire to assess adherence [23]. Among the studies evaluated MDP, one study evaluated traditional MDP score by 17-point questionnaire [36], four studies [35, 44–46] measured MED score by Trichopoulou et al. [48], one study used the MED Quality Index (KIDMED) score [21], one study with three approaches of MDP index, prevention with MED (PREDIMED) score and Trichopoulou's MED score [23] and one with relative “Mediterranean” diet score (rMED) method [34].

The quality assessment of the included studies is presented in Table 4. The quality total score of studies ranged between 6 and 9 while most of them had medium and strong qualities scored by NOS scaling method. Based on our search of the literature, no prior study has assessed the relationship between other dietary patterns such as the Asian dietary patterns (traditional "Japanese" and "Chinese" diets and etc.), "Nordic", or "French" diets and HRQOL. In summary among thirteen included studies, ten studies found significant relations between dietary patterns (dietary patterns derived with factor or cluster analysis or MDP) and HRQOL while three studies did not observe any significant relations between dietary patterns and HRQOL [21–23]. Because of the great heterogeneity between the methodological approaches, study designs and report of results, the data synthesis and meta-analysis was not possible.

**Table 4:** Newcastle–Ottawa Quality Assessment Scale (NOS) for studies included in the systematic review

Author name	Study design	Selection				Comparability	Outcome		
		Representativeness of the sample	Sample size	Non-respondents	Ascertainment of the exposure		Assessment of the outcome	Statistical test	Final score
Galilea-Zabalza et al. [36]	Cross-sectional	*	–	*	*	*	**	*	8
Bonaccio et al. [35]	Cross-sectional	*	*	*	*	**	**	*	9
Milte et al. [46]	Cross-sectional	*	*	*	*	**	**	*	9
Alcubierre et al. [34]	Cross-sectional	*	*	**-**	*	*	**	*	7
Holmes et al. [22]	Cross-sectional	*	*	**-**	**-**	*	**	*	6
Kim et al. [17]	Cross-sectional	*	*	*	*	**	**	*	9
Zaragoza-Marti et al. [45]	Cross-sectional	*	*	-	*	*	*	*	6
Moravejolahkami et al. [47]	Cross-sectional	*	*	*	*	**	**	*	9
Mozzillo et al. [21]	Cross-sectional	*	*	*	*	**	**	*	9

Author name	Study design	Selection				Comparability	Outcome			
		Representativeness of the exposed cohort	Selection of the non-exposed cohort	Ascertainment of exposure	Demonstration that outcome of interest was not present at start of study		Assessment of outcome	Was follow-up long enough for outcomes to occur	Adequacy of follow up of cohorts	Final score
Ruano et al. [28]	Cohort	*	*	*	*	**	*	*	*	9
Perez-Tasigchana et al. [23]	Cohort	*	*	*	*	**	*	*	*	9
Perez-Tasigchana et al. [23]	Cohort	*	*	*	*	**	*	*	*	9
Gigic et al. [27]	Longitudinal	*	*	*	*	**	*	*	*	9
Sanchez-Aguadero et al. [44]	Longitudinal	*	*	*	*	**	*	*	*	9

One star represents a score of 1, and a study can be awarded a maximum score of 9 in total. The items were scored “*”if the answer was “YES,” and “−” if the answer was “NO” or “UNCLEAR.” The final quality scores were as follows: low quality = 0–3; moderate quality = 4–6; high quality ≥ 7

## Discussion

### Principal findings

In the current systematic review, the studies reporting the relationship between dietary patterns and HRQOL were reviewed. Most of the included studies showed the relationship between dietary patterns and HRQOL with only three exceptions showing no association in subjects with minor digestive problems and type1 diabetes [21–23]. Of the studies evaluating the dietary patterns reported higher adherence to healthy dietary patterns are associated with better scores of HRQOL in one or more dimensions [17, 27, 28, 46, 47]. Also, higher adherence to MDP was associated with better scores of HRQOL [23, 34–36, 44, 45]. To our knowledge, this is the first systematic review to examine the effect of dietary patterns on HRQOL among general population without age or disease restrictions.

### Details from previous research/studies

To date, only one systematic review has been carried out to investigate the relationship of dietary patterns and quality of life in older people [4]. Like our results, they revealed that healthy dietary patterns were related to better quality of life in one or more domains and adherence to MED were significantly related to improvement in at least one of the quality of life domains. In another study by Ruano et al. [28] evaluating the baseline data of the SUN cohort, two major dietary patterns of the "Western" dietary pattern (rich in processed pastries and red meats) and the MDP (high in olive oil, vegetables and fruits) was identified among 11,128 participants. The "Western" dietary pattern was inversely associated with all domains of HRQOL. The magnitude of these differences between the participants in the highest versus lowest quintile of adherence to the "Western" dietary pattern ranged from 20.8 (for mental health) to 23.5 (for vitality). In opposite, the MDP was associated with better HRQOL domains while scores ranged from + 1.3 (for physical functioning) to + 3.4 (for vitality). In the study by Kim et al. [17] and Gigic et al. [27] among patients with colorectal and breast cancer, patients with higher adherence to "Western" diet had lower chances to improve in physical functioning, diarrhea and constipation. While, patients following a "Fruit and vegetable" and "Healthy" diet indicated improving diarrhea and dyspnea scores.

The deleterious effects of "Western" dietary patterns have been widely mentioned in numerous studies; its association with metabolic syndrome, obesity, insulin resistance [49], and risk of cardiovascular disease [50, 51]. Moreover, considering the relationship between "Western" dietary pattern and mental component summary of HRQOL, some studies reported an inverse relationship between "Western" dietary pattern and depression, anxiety [52], mental [53] and cognitive disorders [54]. One possible explanation is that high in saturated and trans fats, refined sugars, red and processed meat could reduce the brain derived neurotropic factor (BDNF) concentrations and to inhibit its expression via several pro-inflammatory cytokine production [55, 56].

In the study of Moravejolahkami et al. [47], examining the association between dietary patterns and HRQOL among 261 multiple sclerosis patients, results showed that ''Fruits, vegetables, low fat dairy-based'' pattern and ''Mediterranean-Like'' dietary pattern were associated with higher physical and mental health composite scores (P adjusted < 0.001). In another study by Sanchez-Aguadero et al. [44], greater adherence to the MED was related to higher scores on the SF-12 social functioning and mental component. After adjustment for confounders, for each point of increase in the MED adherence score, there was an increase of 1.17 points in the mental component value (p < 0.01). It is well documented that MDP is potentially able to protect against numerous chronic disease including cardiovascular events [57, 58] metabolic syndrome [59] and diabetes [60]. Also, from mental component summary point of view, MED could potentially decrease the risk of depression [61] cognitive decline and dementia [62, 63]. Higher consumption of vegetables and fruits as a characteristic of MDP is associated with improved HRQOL [64]. Therefore, it is not out of expected that MED is related to improve in both mental and physical domain of HRQOL.

In addition, less than 25% of the studies (3 out of 13 studies) included in this review reported no significant association between dietary patterns and HRQOL. Mozzillo et al. [21] fails to show any significant relationship between adherence to the MED and HRQOL, maybe due to the low number of patients with poor diet quality in their study. Perez-Tasigchana et al. [23] found that PREDIMED scores were related to a marginally better PCS in the Seniors-ENRICA cohort and also reported no association between MED score and any of the HRQOL domains in the UAM-cohort. The studies were performed 10 years apart and both the studies used different methods to measure dietary pattern and HRQOL, and found consistent findings. In a cross sectional study by Holmes et al. [22]; it was noted that no significant differences in HRQOL were found between dietary patterns. They used cluster analysis to derive dietary patterns and FBA questionnaire for assessment of HRQOL.

## Summary

There is an obvious fact that existing studies on dietary patterns focus mainly on MED or "Western" dietary patterns, and very little attention has been given to the effect of the other dietary patterns such as the "Japanese", "Nordic", "French", or "Chinese" traditional diet on HRQOL and more studies are required on these neglected dietary patterns. Accordingly, our study focuses more on MDP and "Western" dietary patterns. The "Nordic" diet is characterized by high intake of root vegetables, grains, berries, nuts, and seafood. The "Nordic" diet is similar to the MED but the "Nordic" diet emphasizes canola oil more than olive oil [65]. A recent cross-sectional study showed that high adherence to the "Nordic" diet was associated with a healthier lifestyle [66]. Side dishes in "Japanese" traditional diet include several species of fish that are a rich source of high quality protein as well as omega-3 acids, which are believed to be beneficial for human health [67].

As reported in the current review, most of the studies regarding the associations between dietary patterns and HRQOL were considered the healthy benefits of MDP in improvement of quality of life. Although there was a great heterogeneity in estimating the MED score, however, almost all of the studies had consistent results reporting the positive effects of the MED on HRQOL. In spite of observed associations between HRQOL and dietary patterns, the possible underlying mechanisms are unclear. In fact, all of the life domains including personal satisfaction, social interactions or economical characteristics could have direct relationships with food behaviors and eating ways and, together with the physical and mental health, could explain what we consider to be a good or poor quality of life [68].

## Strengths and limitations

The current broad systematic review was performed according to PRISMA guidelines and was included all of the studies up to March 2020 in its literature search. We combined the studies in numerous disease and age groups because of the limited number of studies. Moreover, the included studies were large observational studies with cross-sectional design and therefore the causal inference could not be relied. Additionally, the dietary assessments and quality of life were both assessed by self-reported tools and might be a source of bias. Lastly, it will be better to develop a nutrition-specific quality of life assessment tool to better interpretation of the results of the effects of diet on the quality of life. The dietary assessment, quality of life measurements and data visualization varied from one study to another and the great heterogeneity among included studies regarding the design, dietary assessment, quality of life assessment tools and statistics made us unable to run a meta-analysis.

## Conclusion

In conclusion, according to our findings, MDP and "Healthy" dietary patterns are associated with better dimension scores of HRQOL in both physical and mental summaries. While, "Unhealthy" dietary patterns and "Western" dietary patterns are associated with lower scores of HRQOL. Adjusting for the potential confounders, the results might be identifiable for final causal inference. Because of the great heterogeneity between the methodological approaches, designs and report of results, the meta-analysis was not possible. Further longitudinal studies are required to clarify the association between dietary patterns and HRQOL.

## Data Availability

Not applicable.

## Abbreviations

ADDQoL-19: Audit of Diabetes-Dependent Quality of Life
EQ-5D: The European Quality of Life-5 Dimensions
FFQ: Food Frequency Questionnaire
HRQOL: Health-related quality of life
KDQOL-36: Kidney Disease Quality of Life 36-item survey
MANSA: The Manchester Short Appraisal was used to assess self-rated quality of life
MCS: Mental component score
MDP: Mediterranean dietary pattern
MED: Mediterranean diet
MeSH: Medical subject headings
MSQOL-54: Multiple Sclerosis Quality of Life-54 questionnaire
NOS: The Newcastle–Ottawa Scale
PCS: Physical component score
PedsQL 3.0 DM: Pediatric Quality of Life Inventory 3.0 Diabetes Module
PREDIMED: Score prevention with Mediterranean diet score
rMED: Relative Mediterranean diet score
SF-12: The 12-item Short Form
SF-36: The 36-item Short Form
